# Supplementary material for: Classification of periodontitis stage and grade using natural language processing techniques
Source: PLOS Digit Health. 2024 Dec 13;3(12):e0000692. doi: 10.1371/journal.pdig.0000692 (PMC11642968; doi:10.1371/journal.pdig.0000692)
Supplement: Attachment — Submitted filename: Rebuttal letter (PLOS Digital Health).docx [file pdig.0000692.s001.docx]

**Editor’s comment**

The study on periodontitis classification using BERT and RegEx/MLP approaches has several limitations that impact its reliability and generalizability. The small sample size from a single clinic, data imbalance, and lack of comparison with other state-of-the-art methods raise concerns about the model's performance and applicability. Additionally, the paper fails to address important aspects such as model interpretability, overfitting prevention, and ethical considerations in medical AI applications.

**Answer**

Dear Editor,

Thank you for your valuable feedback. As highlighted in our manuscript, research on the application of NLP techniques, including RegEx/MLP, in dentistry—particularly in periodontology—is still in its early stages. Our study is among the first to utilize these methods for classifying periodontitis, and we believe it adds important insights by comparing two different approaches.

However, we acknowledge that our findings should be interpreted with caution due to the preliminary nature of this research. The limited dataset in this study posed a notable challenge, which is a common issue in dental studies where acquiring a large number of samples is often difficult due to factors such as patient availability and referral practices. Future studies should focus on analyzing larger, more balanced datasets and exploring various NLP techniques to further validate our findings. Collaborations across multiple dental centers could also help in pooling data to achieve larger and more representative datasets.

We recognize that data imbalance was a notable challenge in our study, with most of the training data representing patients in stage III and grade B periodontitis. This imbalance, as also noted in the study by Alawaji et al., (Alawaji YN, Alshammari A, Mostafa N, Carvalho RM, Aleksejuniene J. Periodontal disease prevalence, extent, and risk associations in untreated individuals. Clin Exp Dent Res. 2022 Feb;8(1):380-394. doi: 10.1002/cre2.526. Epub 2022 Jan 10. PMID: 35015383; PMCID: PMC8874091) reflects the common referral pattern where the majority of patients present with moderate to severe periodontitis. Such imbalances can lead to models that are biased towards predicting more prevalent categories, potentially reducing their effectiveness in identifying less common conditions. To address this, we computed class weights during BERT training and assessed model performance on a new set of patient notes to evaluate generalizability. Regularization techniques, such as dropout, were also employed during training to mitigate overfitting.

Despite these challenges, the results of our research have clinical relevance. The accuracies observed, particularly in advanced stages of periodontitis, suggest that these methods can offer reliable diagnostic support, even with unbalanced datasets. Clinically, this implies that AI tools can assist in identifying severe cases that require immediate intervention, thereby improving patient management and outcomes.

For comparison, the study by Oh et al. (2021) on periodontitis classification among dental practitioners with different educational backgrounds reported lower accuracy levels compared to our study. Our methods demonstrated higher accuracy in determining the stage and grade of periodontitis than those observed with human evaluators in Oh et al.'s study, highlighting the potential of AI in enhancing diagnostic consistency.

Furthermore, we acknowledge the importance of ethical considerations in the development and application of medical AI. Ensuring that AI models are developed and tested in a manner that respects patient privacy, avoids bias, and enhances equitable healthcare delivery is crucial. Our study adhered to ethical standards by anonymizing patient data and focusing on model transparency.

A more detailed explanation of the models and techniques has been added to the revised version of the manuscript (Methods section).

**Reviewer #1**

1- The study used only 309 patient records from a single university clinic. This small, potentially homogeneous sample may not be representative of the broader population, limiting generalizability.

**Answer:**

Thank you for your valuable feedback. We recognize that our findings should be interpreted with caution given the preliminary nature of this research. The limited dataset posed a significant constraint, as collecting a large number of samples in dental studies can be challenging due to factors such as patient availability and referral practices (Schwendicke et al., 2020). However, in this study, we reviewed 1513 patients’ charts and only 309 met the criteria for data availability. Future research should aim to analyze larger, more balanced datasets and explore different NLP techniques to further validate our results. Collaborations across multiple dental centers could also facilitate data pooling, resulting in larger and more representative datasets.

2- The authors noted an imbalance in the input data, with more patients in stage III and grade B. This can lead to biased model performance.

**Answer:**

We acknowledge that data imbalance posed a significant challenge in our study, as the majority of the training data consisted of patients with stage III and grade B periodontitis. This imbalance, which is consistent with the referral patterns observed in the study by Alawaji et al. (Alawaji et al., 2022), reflects the tendency for patients to present with moderate to severe periodontitis. Such imbalances can cause models to be biased toward predicting more common categories, potentially limiting their effectiveness in detecting less frequent conditions. To address this issue, we adjusted class weights during BERT training and evaluated the model's performance on a new set of patient notes to assess generalizability. Additionally, regularization techniques like dropout were applied during training to reduce the risk of overfitting. This information has been added to the manuscript “methods” section.

3- While the study compared BERT with a RegEx/MLP approach, it didn't explore other state-of-the-art NLP or machine learning methods.

**Answer:**

Thank you for your valuable feedback. As highlighted in our manuscript, research on applying NLP techniques, including RegEx/MLP, in dentistry—particularly in periodontology—is still in its early stages. Our study is among the first to utilize these methods for classifying periodontitis, and we believe it adds important insights by comparing two different approaches.

Our study focused on comparing BERT with the RegEx/MLP approach and did not explore other state-of-the-art NLP or machine learning methods. This choice was intentional, as we aimed to establish a foundational understanding of these specific methods in this novel application. Given the limited research in this area, we prioritized a detailed analysis of these approaches to set a baseline for future studies. Other approaches such as large language models, topic modeling and latent Dirichlet allocation were all considered but did not fit with the given size of the data used in this model.

That said, we agree that future research should explore additional NLP models, such as other transformer-based models or traditional machine learning techniques, to provide a more comprehensive comparison. Incorporating these methods in future work will help advance the development of more effective tools for periodontitis classification.

4- The study doesn't provide insight into which features or text patterns are most important for classification. Incorporate model interpretability techniques like SHAP values or LIME.

**Answer:** We have provided examples of probabilistic scoring approaches using LIME (Figure 1) but they serve as an illustration of the method with text examples, but does not provide explanatory information for how it is considered as it just highlights which word was most probabilistic in the differentiation of categories.

5- The paper doesn't provide a detailed analysis of misclassified cases.

**Answer:**

Thank you for the comment. Two figures representing the confusion matrices for stage and grade classifications, comparing BERT with RegEx/MLP, along with the appropriate explanations, have been included in the manuscript.

6- While the models show good statistical performance, there's no indication of how well they align with expert clinical judgment.

**Answer:**

The below text has been added to the “Discussion” comparing the performance of our proposed models with those of clinicians:

“The study by Oh et al. (2021) revealed that dental practitioners with periodontal backgrounds had higher accuracy in classifying periodontitis stages (71.33%) compared to grades (64%), with non-periodontal practitioners showing a similar trend but with lower accuracy (61.67% for stages and 49.33% for grades). In contrast, our BERT model demonstrated higher accuracy in predicting grades (72%) than stages (66%) for new patients, with these results being closer to the accuracy levels achieved by periodontal specialists. Additionally, the BERT model's classification accuracy for both stage and grade surpasses that of non-periodontal practitioners, while the MLP model, with an accuracy of 62.5% for grades and 59.4% for stages, also outperforms non-periodontal clinicians. These comparisons suggest that our BERT model aligns more closely with specialist performance but both approaches exceed the accuracy of non-specialists in classifying the periodontitis stage and grade, indicating their strong potential for effective clinical application in the disease classification.”

7- The study doesn't mention techniques used to prevent overfitting, especially given the small dataset.

**Answer:**

We have added the below texts to the “methods” section: “dropout layers with ReLU activation functions. These layers enabled the model to learn higher-level features and prevent overfitting by introducing regularization” and “Additionally, early stopping was implemented as a regularization technique to prevent overfitting based on validation loss. Through monitoring the validation loss, the early stopping mechanism terminates training when the model's performance on unseen data begins to deteriorate. By restoring the best weights observed during training, this approach helps to optimize the model's generalization ability and improve its performance on unseen data.”

This was not included previously as we were concerned about article length and readability of the paper.

8- The paper doesn't address potential ethical concerns of using AI for medical diagnosis.

**Answer:**

This is a very important point. However, we are simply trying to demonstrate that the complex process of classification is replicable with the application of text mining method such as BERT. This tool, while important in demonstrating the replicability of medical diagnosis, we are not proposing applications of AI for medical diagnosis.

We have added the below paragraphs in the “discussion” section:

“However, the ethical concerns surrounding AI in medical diagnosis, particularly issues related to bias, transparency, and patient privacy, have led to restrictions on access to large, diverse datasets in healthcare. This limited access exacerbates the challenge of developing unbiased and accurate AI models, as the scarcity of data hinders the ability to train AI systems effectively. Without comprehensive datasets, AI models risk perpetuating existing disparities in healthcare, as they may not adequately represent all patient populations. Addressing these ethical challenges is essential to balance the need for robust AI development with the protection of patient rights and the promotion of fair, transparent medical practices.

In spite of access to few numbers of data in our study, and the novelty of our proposed method, our findings showed a relatively high accuracy in correct classification of the stage and grade of the periodontitis, which suggests that our proposed method was successful in extracting the relevant information from patient notes and classifying the stage and grade of the periodontal disease.”

9- The paper lacks some important details about model architecture, hyperparameters, and training procedures.

**Answer:**

A more detailed explanation of the models and techniques has been added to the revised version of the manuscript (Methods section).

Reviewer #2:


1- How difficult it is to determine stage and grade manually? Does the EDR notes usually contain the stage and grade information already? 

**Answer:**

Thank you for your valuable comment. As demonstrated in a study by Oh et al. (2021), dental practitioners with periodontal backgrounds achieved an accuracy of 71.33% for classifying periodontitis stages and 64% for grades, while non-periodontal practitioners had lower accuracy rates of 61.67% for stages and 49.33% for grades. Despite having only 309 patient charts, our proposed models outperformed general practitioners in accurately classifying the disease, with the BERT model aligning closely with the accuracy of periodontists. The EDR notes typically include information on patients' periodontitis stages and grades, serving as a crucial reference for tracking disease progression and anticipating future treatment needs.

2- The technical details in the paper were missing, which makes is very difficult to evalute the paper. For example, what is the exact regular expression used to extract information? Which BERT model was used as base model for fine tuning? How were LSTM combined with BERT?

**Answer:**

Detailed explanation of both methods has been added to the “methods” section. This was not included as we were concerned with word count and readability.

3- Why not use BioBERT or MedBERT as base model?

**Answer:**

Thank you for your insightful comment. We chose not to use BioBERT or MedBERT as the base models because the notes utilized in our study were primarily narrative texts without extensive specialized terminology. BioBERT and MedBERT are specifically designed for processing medical and biomedical data, making them more suitable for tasks that involve a higher concentration of specialized medical vocabulary. However, given the nature of the dental notes in our dataset, a general model like BERT was more appropriate and effective for capturing the context present in the narrative descriptions used in dentistry.

4- Can RegEx accurately extract the information from notes and charts? It is not clear to me why regular expression can be used to extract information from chart? What is the main bottleneck of the RegEx features based MLP? RegEx part or MLP part?

**Answer:**

Thank you for your questions. To clarify, RegEx was specifically used to extract information from the narrative notes, not from the charts (edited in the manuscript). The narrative notes contained key details that were necessary for the classification task, and RegEx proved effective in accurately identifying and extracting these relevant pieces of information. The data from the charts were then combined with the information extracted from the notes to serve as input for the MLP model, which was responsible for classifying the stage and grade of the disease.

As for the bottleneck in the RegEx features-based MLP, it largely depends on the complexity of the information extraction task and the quality of the input data. The RegEx part could be a bottleneck if the narrative notes are inconsistent or contain ambiguous language that makes it difficult to define accurate patterns. On the other hand, the MLP part might be the limiting factor if the model is not adequately trained or if the input features extracted by RegEx do not capture enough information to make accurate classifications. In our case, both components are critical, but the main challenge lies in ensuring that RegEx accurately extracts the necessary details from the notes to provide the MLP with reliable input data.

5- Can the authors show distributions of import statistics of the dataset? e.g. how many datapoint are in stage I, II, III, and grade A, B, C?

**Answer:**

The explanation below has been added: “The prevalence of different periodontitis stage and grades in the collected notes were as follows: stage I= 6, stage II= 36, stage III= 206, stage IV= 61, and grade A= 17, grade B= 202, grade C= 87.”

6- Is there a reason why only stage III and grade B have good performance?

**Answer:**

Thank you for your observation. The reason why stage III and grade B show better performance in our models is likely due to the distribution of the training data. A significant portion of the data was concentrated in these classes, allowing the models to learn and generalize better for these specific categories. This also can be explained by the fact that the majority of patients referred to periodontal clinics (78.4%) have moderate to severe periodontitis (Alawaji et al., 2022).

7- Before branding the proposed the model as diagnostic tool, there are so many issues that needs to be solved. For instance, would the model be used only after being manually diagnosis of periodontitis? Is there any negative data being used in the study (e.g. data from other close disease or healthy patient)?

**Answer:**

Thank you for your insightful feedback. Our proposed models are not intended to replace the clinical judgment of experienced practitioners but to serve as a valuable support tool, particularly for general practitioners or those with limited experience in diagnosing periodontitis. While the manual diagnosis of periodontitis typically requires detailed tooth-by-tooth charting and multiple clinical examinations, our models can diagnose based on narrative notes that include the patient’s medical and dental histories, relevant health issues, and overall history of the disease. Although these notes provide substantial context, they do not include detailed data such as probing depths or specific clinical examination results. Also, the models effectively integrate key factors like systemic diseases, such as diabetes and heart conditions, which are known to influence periodontitis progression. These conditions were included in the patients' notes and were considered in the models, making it a comprehensive tool for supporting clinical decisions. However, it should be noted that the study did not include negative data from other diseases or healthy patients, as the focus was on accurately classifying periodontitis stages and grades. This integration ensures the model offers a more accessible and less invasive means of assessing periodontitis, especially for practitioners needing additional diagnostic support.

8- Do the authors consider using existing large language models (LLM) for zero or few shot learning? LLMs are a good baseline to compare with the propose model. For instance, with proper prompt engineering for Llama3 or GPT4 by including the standard diagnostic standard as system prompt and the EDR notes as user prompt. In addition, even for the task of extracting information from EDR, LLM should be better than RegEx as RegEx does not understand sematic information.

**Answer:**

Thank you for your insightful comment. While using LLMs is a promising approach, our focus was on developing a model specifically tailored to dental records and periodontitis classification, given that our primary audience is dentists and clinicians. LLMs could serve as strong baselines, particularly in general medical contexts. However, the specialized nature of dental terminology and the specific needs of our audience led us to use RegEx for its simplicity and effectiveness in extracting structured information from narrative notes. Although LLMs offer better semantic understanding, RegEx and specifically, BERT met our needs in this context. We acknowledge the potential of LLMs for enhancing the extraction process and plan to explore this in future work to compare their effectiveness with our custom models.

9- Can the authors comment on how robust the model is? What will happen if the model was applied to notes that is written by another dentists?

**Answer:**

Thank you for your comment. To address concerns about the robustness of the model, it's important to note that the dataset we used included notes written by different dentists working in the clinic, not just a single individual. This diversity in the notes helped the model to learn from a variety of writing styles and terminologies, making it more robust and better equipped to handle variations in documentation. Therefore, while there may still be some impact on performance if applied to notes from entirely new practitioners, the model has already been exposed to a range of writing styles, which should help mitigate this issue. Nonetheless, continuous validation with new data will be essential to ensure consistent performance across different contexts.
