# Supplementary material for: Classification of periodontitis stage and grade using natural language processing techniques
Source: PLOS Digit Health. 2024 Dec 13;3(12):e0000692. doi: 10.1371/journal.pdig.0000692 (PMC11642968; doi:10.1371/journal.pdig.0000692)
Supplement: Attachment — Submitted filename: 2nd Rebuttal letter.docx [file pdig.0000692.s002.docx]

The reviewers suggest additional several areas for improvement and further exploration.

1- They recommend incorporating model interpretability techniques, providing a detailed analysis of misclassified cases, and addressing potential overfitting issues.

The analysis of the misclassification included in the “Results” as below:

“During the evaluation, we closely analyzed the misclassified cases to identify patterns or common features leading to incorrect predictions. It was observed that a significant portion of misclassified cases belonged to the less prevalent stages and grades, indicating a potential data imbalance issue. This analysis highlights the need for enhancing data augmentation techniques to better represent underrepresented classes in the dataset. According to Figure 2, the RegEx/MLP model generally performs better in reducing misclassifications, particularly for stage III cases, where it correctly identifies 17 out of 21 instances compared to BERT's 14. However, the BERT model exhibits slightly fewer misclassifications overall, especially for Stage IV cases, and handles stage I classifications well, with minimal confusion, indicating higher reliability in identifying early-stage cases (Figure 2).

The comparison between the BERT and RegEx/MLP models in the context of grade classification on the new unseen data, as illustrated in Figure 3, shows that both models perform similarly in correctly identifying grade B cases, with each model correctly classifying 18 instances. However, the BERT model exhibits a slightly better performance in reducing misclassifications for grade C cases, where it correctly classifies 5 out of 9 instances, compared to the RegEx/MLP model’s 2. Both models show difficulty in correctly identifying grade A cases, with BERT misclassifying both instances as grade B and RegEx/MLP misclassifying them as grade C (Figure 3).”

To address the model interpretability potential, the paragraph below is included in the “Methods” section:

“We implemented model interpretability techniques such as Local Interpretable Model-Agnostic Explanations (LIME) to analyze the predictions made by the BERT model. LIME helps in identifying the key features that influence the model's decision-making process for classifying periodontitis stages and grades. This interpretability approach allows us to better understand the reasoning behind correct and incorrect classifications, providing insights into the strengths and weaknesses of the model. However, it is important to note that the primary aim of our study was not to conduct a detailed analysis of the specific features driving the predictions but to demonstrate the overall classification accuracy and applicability of these models for periodontal staging and grading.”

To address potential overfitting issue, the explanation below is included in the “Discussion”:

“The model demonstrated higher accuracy in classifying stage III and grade B periodontitis, which can be attributed to the imbalanced nature of the input data, with a greater number of patients falling into these categories compared to stages I or II and grade A. This imbalance is likely due to the increased number of referrals for patients with more severe periodontal conditions. Despite the relatively small sample size of 309 patients—a recognized limitation for a ML study—our findings indicate a relatively high accuracy in classifying the stage and grade of periodontitis. It is crucial to acknowledge that this class imbalance, with a predominance of patients in stage III and grade B, inherently influenced the model’s performance, partly due to the higher referral rates for severe cases. The model's ability to achieve higher accuracy in these classes, even with a limited sample size, underscores its robustness in handling imbalanced data. To address potential overfitting, we incorporated regularization techniques, including dropout layers and early stopping, in the model training process. Despite these measures, a slight tendency towards overfitting was observed, particularly in the later stages of training.”

2- Additionally, they advise exploring the alignment between model performance and expert clinical judgment.

Thank you for your valuable feedback. In our study, we compared the model's predictions to expert judgments, which served as the ground truth. This approach ensured that the evaluations were aligned with clinical expertise, enhancing the reliability and validity of the results. We believe that this comparison to expert assessments further highlights the model's capability in detecting and classifying periodontal disease stages and grades. We will include this point in the revised manuscript to clarify the robustness of our ground truth data. Also, the below paragraphs are added to “Discussion” to compare the model’s performance with traditional methods and clinical experts:

“In comparison to traditional clinical methods and other published approaches for

periodontal staging and grading, our BERT model demonstrated promising results by just

utilizing clinical notes without any imaging data. In contrast to Ertas et al [44], our study

focused on using textual data from patient notes, which is often more readily available in

clinical settings compared to comprehensive radiographic data. Our BERT model

achieved an accuracy of 72% for grading and 66% for staging, which aligns closely with

the performance levels of periodontal specialists observed by Oh et al., who reported

accuracies of 71.33% for staging and 64% for grading among clinicians with periodontal

backgrounds [51] This suggests that the use of NLP techniques like BERT can potentially

bridge the gap between non-specialists and specialists in clinical diagnosis.

Additionally, in a recent study by Tastan Eroglu et al. [52], the ChatGPT model was used to classify periodontitis based on textual inputs, with staging and grading accuracies of 59.5% and 50.5%, respectively. These values are lower compared to our BERT and even RegEx models’ performance, highlighting BERT's superiority in understanding clinical text for periodontitis classification. This difference may be attributed to BERT's fine-tuning capabilities on domain-specific data, which enables it to capture more nuanced clinical features compared to general-purpose models like ChatGPT.

These comparisons indicate that while the BERT model may not yet match the highest

accuracies achieved with image-based methods for staging, its performance is superior for text-based classification tasks. This demonstrates its potential utility in settings where detailed radiographic data may not be available, making it a valuable tool for enhancing decision support in clinical practice. However, future studies should aim to integrate multimodal data—combining both textual and radiographic information—to leverage the strengths of each data type and further improve classification accuracy.”

3- Addressing ethical concerns related to AI in medical diagnosis

Thanks for the comment. The below paragraph is added in the “Discussion” to address

the ethical concerns related to AI in dental diagnosis:

“However, the ethical concerns surrounding AI in medical diagnosis, particularly issues related to bias, transparency, and patient privacy, have led to restrictions on access to large, diverse datasets in healthcare [46, 56]. This limited access exacerbates the challenge of developing unbiased and accurate AI models, as the scarcity of data hinders the ability to train AI systems effectively. Without comprehensive datasets, AI models risk perpetuating existing disparities in healthcare, as they may not adequately represent all patient populations [57]. Addressing these ethical challenges is essential to balance the need for robust AI development with the protection of patient rights and the promotion of fair, transparent medical practices.”

4- Including more details about model architecture, hyperparameters, and training procedures.

To address this concern, Table 1 is provided in the “Methods”, which contain information about model hyperparameters and architecture.

Table 1. Architecture and hyperparameters for the BERT model

| Layer | Parameter Name | Candidate Values | Selected Value |
| --- | --- | --- | --- |
| BERT | Number of parameters | 110M | 110M |
|  | Transformer blocks | 12 layers | 12 layers |
|  | Attention heads | 12 | 12 |
|  | Hidden neurons | 768 | 768 |
| Dropout | Dropout rate | 0.2-0.5 | 0.2 |
| LSTM | Decay Rate | 0.96-0.97 | 0.97 |
|  | Activation Function | ReLU, sigmoid | ReLU |
|  | Learning Rate | 0.1-10e-7 | 5e-6 |
|  | Momentum | 0.5-0.9 | 0.5 |
| Dense | Neurons | 25-100 | 64 |
| Model compile | Epoch | 15-50 | 40 |
|  | Batch Size | 12-36 | 20 |

**Reviewer #1:**

Overall, the manuscript shows notable improvement in terms of technical detail and transparency. However, there are still some important considerations around clinical applicability and broader context that could be addressed to strengthen the paper further. The core contribution - applying BERT to periodontal staging and grading - remains novel and potentially impactful, but the limitations of the current study should be clearly communicated.  
Major comments

1- While acknowledged as a limitation, the relatively small sample size (309 patients) is still a significant concern for a machine learning study, especially given the class imbalance.

While we acknowledge that the sample size of 309 patients presents a limitation, it's important to emphasize that this study represents one of the first attempts to apply BERT-based models to classify periodontitis stage and grade using clinical notes in the field of dentistry. Despite the limited sample size, our model's performance demonstrates significant promise, achieving an accuracy of 77% for stage prediction and 75% for grade prediction, which is higher than the accuracy of the baseline MLP model (59.4% for stage and 62.5% for grade) on a similar dataset.

Additionally, we applied strategies to address the class imbalance in our dataset, such as using the class_weight to ensure that the model assigned appropriate importance to each class during optimization. This approach helps to mitigate the effect of imbalanced data distributions, enhancing the model's robustness even with a relatively small and imbalanced dataset.

When compared to clinical experts, our model's performance is even more encouraging. The study by Oh et al. revealed that dental practitioners with periodontal backgrounds had higher accuracy in classifying periodontitis stages (71.33%) compared to grades (64%), with non-periodontal practitioners showing a similar trend but with lower accuracy (61.67% for stages and 49.33% for grades). In contrast, our BERT model demonstrated higher accuracy in predicting grades (72%) than stages (66%) for new patients, with these results being closer to the accuracy levels achieved by periodontal specialists. Additionally, the BERT model's classification accuracy for both stage and grade found to be higher compared to those made by non-periodontal practitioners, while the MLP model, with an accuracy of 62.5% for grades and 59.4% for stages, also outperforms non-periodontal clinicians. These comparisons suggest that our BERT model aligns more closely with specialist performance, but both approaches exceed the accuracy of non-specialists in classifying the periodontitis stage and grade, indicating their strong potential for effective clinical application in disease classification.

While we recognize that a larger and more balanced dataset could further improve the model's performance and generalizability, the ethical and practical challenges in obtaining large-scale clinical data in healthcare settings must also be considered. We believe that this study lays the groundwork for future research, and we recommend that subsequent studies build on this work by including more diverse and larger datasets to validate the findings.

In response to your comment, we have included the text below in the “Discussion” for more clarification:

“The model demonstrated higher accuracy in classifying stage III and grade B periodontitis, which can be attributed to the imbalanced nature of the input data, with a greater number of patients falling into these categories compared to stages I or II and grade A. This imbalance is likely due to the increased number of referrals for patients with more severe periodontal conditions. Despite the relatively small sample size of 309 patients—a recognized limitation for a ML study—our findings indicate a relatively high accuracy in classifying the stage and grade of periodontitis. It is crucial to acknowledge that this class imbalance, with a predominance of patients in stage III and grade B, inherently influenced the model’s performance, partly due to the higher referral rates for severe cases. The model's ability to achieve higher accuracy in these classes, even with a limited sample size, underscores its robustness in handling imbalanced data. To address potential overfitting, we incorporated regularization techniques, including dropout layers and early stopping, in the model training process. Despite these measures, a slight tendency towards overfitting was observed, particularly in the later stages of training.”

2- While LIME analysis is mentioned, there could be more in-depth discussion of what features the models are using to make predictions and how this aligns with clinical understanding.

Thanks for your insightful comment. To address this comment regarding the LIME analysis and provide more in-depth discussion on the features used by the models, we will highlight that the primary focus of the study was not on identifying specific features but rather on evaluating the model's overall performance in classifying periodontitis. It is important to note that the primary aim of our study was not to conduct a detailed analysis of the specific features driving the predictions but to demonstrate the overall classification accuracy and applicability of these models for periodontal staging and grading. Our focus was on assessing the models' ability to classify periodontitis stages and grades using unstructured patient notes, rather than explicitly identifying individual features or variables contributing to each decision. The LIME analysis provided a way to visualize the model's decision-making process, which helps clarify how the model arrives at its conclusions, but this was not the central aspect of our investigation.

We have clarified in the “Results” that the LIME analysis was included to provide some interpretability to the results and to make them more understandable for the readers, but due to word count limitations, we could not elaborate further on this topic. The added paragraph is:

“We implemented model interpretability techniques such as Local Interpretable Model-Agnostic Explanations (LIME) to analyze the predictions made by the BERT model. LIME helps in identifying the key features that influence the model's decision-making process for classifying periodontitis stages and grades. This interpretability approach allows us to better understand the reasoning behind correct and incorrect classifications, providing insights into the strengths and weaknesses of the model. However, it is important to note that the primary aim of our study was not to conduct a detailed analysis of the specific features driving the predictions but to demonstrate the overall classification accuracy and applicability of these models for periodontal staging and grading.”

3- The discussion could be expanded to more clearly articulate how these models could be integrated into clinical workflows and what potential benefits/risks this might entail.

Thanks for your comment. To address the comment and expand on the discussion, we have added specific explanations about integrating these models into clinical workflows and their potential benefits and risks as below:

“Integrating these AI models into clinical workflows could enable real-time analysis of patient data directly from digital records, providing immediate diagnostic support for clinicians. This integration would streamline the diagnostic process, reduce the need for additional tests, and potentially lower costs. However, challenges such as data standardization, interoperability among EDR systems, and clinician training must be addressed to ensure seamless integration. Risks include the possibility of over-reliance on AI predictions, which might lead to misdiagnosis if the model's limitations are not well understood or if data quality issues are not adequately managed.”

“The integration of ML and DL models into clinical workflows has the potential to transform periodontal diagnosis by providing a decision-support tool that complements clinical expertise. These AI models can assist clinicians in quickly analyzing large volumes of patient data, identifying disease patterns, and generating treatment recommendations based on real-time information. However, risks include potential data biases, ethical concerns regarding patient privacy, and the need for transparent AI decision-making processes to avoid unintended consequences in clinical settings. Future studies should explore strategies to mitigate these risks while maximizing the benefits of AI-driven diagnostics in periodontal care.”

4- While the study compares BERT to RegEx/MLP, it would be valuable to see how these methods compare to current clinical practice or other published approaches for periodontal staging and grading.

Thank you for your comment. To make a comparison with current clinical practice and recent published methods, the section below is included in the “Discussion”:

“In comparison to traditional clinical methods and other published approaches for periodontal staging and grading, our BERT model demonstrated promising results by just utilizing clinical notes without any imaging data. In contrast to Ertas et al [44], our study focused on using textual data from patient notes, which is often more readily available in clinical settings compared to comprehensive radiographic data. Our BERT model achieved an accuracy of 72% for grading and 66% for staging, which aligns closely with the performance levels of periodontal specialists observed by Oh et al., who reported accuracies of 71.33% for staging and 64% for grading among clinicians with periodontal backgrounds [51] This suggests that the use of NLP techniques like BERT can potentially bridge the gap between non-specialists and specialists in clinical diagnosis.

Additionally, in a recent study by Tastan Eroglu et al. [52], the ChatGPT model was used to classify periodontitis based on textual inputs, with staging and grading accuracies of 59.5% and 50.5%, respectively. These values are lower compared to our BERT and even RegEx models’ performance, highlighting BERT's superiority in understanding clinical text for periodontitis classification. This difference may be attributed to BERT's fine-tuning capabilities on domain-specific data, which enables it to capture more nuanced clinical features compared to general-purpose models like ChatGPT.

These comparisons indicate that while the BERT model may not yet match the highest accuracies achieved with image-based methods for staging, its performance is superior for text-based classification tasks. This demonstrates its potential utility in settings where detailed radiographic data may not be available, making it a valuable tool for enhancing decision support in clinical practice. However, future studies should aim to integrate multimodal data—combining both textual and radiographic information—to leverage the strengths of each data type and further improve classification accuracy.”

5- The conclusion could be strengthened by more specific suggestions for future research to address the current limitations.

Thank you for your insightful comment. We have included the below explanations to address this comment:

“Specifically, exploring techniques such as synthetic data generation and data augmentation could be valuable in overcoming the challenge of class imbalance and enhancing model performance. Moreover, evaluating the integration of other machine learning approaches, like ensemble methods, may further improve classification accuracy.”

“Future research should focus on longitudinal studies to assess the predictive value of these models over time, along with a detailed comparison of different NLP and DL architectures to identify the most efficient approaches for clinical implementation.”

“It is recommended that future studies compare various BERT models and other NLP techniques to identify the most effective ones for extracting information from EDR to improve classification accuracy and clinical applicability.”
